# Supplementary material for: Infection with Jujube Witches’ Broom Phytoplasma Alters the Expression Pattern of the Argonaute Gene Family in Ziziphus jujuba
Source: Microorganisms. 2025 Mar 14;13(3):658. doi: 10.3390/microorganisms13030658 (PMC11944418; doi:10.3390/microorganisms13030658)
Supplement: Supplementary file 1 [file microorganisms-13-00658-s001.zip › Table S1.pdf]

Table S1. Primer design for qRT-PCR assay

Gene name	Primer Sequence (5′-3′)
ZjAGO1	Forward: TTCAGCACCAAGCAACTTCC<br>Reverse: ACAAGAAGCCTTGCAGTTCG
ZjAGO2	Forward: ACAAGAAGGTGGACCTAGCC<br>Reverse: CCCTTGATGTGACCTCTGGT
ZjAGO3	Forward: TACGTGGCACAGTTAGTGGT<br>Reverse: CAGAGATGCACAAGCCTTCC
ZjAGO4	Forward: GCTTGGAGGTAGCAATGTGG<br>Reverse: ATTTGCAGCAGGCCATTTCA
ZjAGO5	Forward: ACTTGTTTCACTGCAACGCT<br>Reverse: CACGGCCATTGACTTGAGTT
ZjAGO6	Forward: CGCTGGTGGAGAAATCAAGG<br>Reverse: TCAACCTAGGAGCAGCCAAA
ZjAGO7	Forward: ACGATTAGGCCAGAGGTCAC<br>Reverse: GGAAGAGGCATCGTCAGGTA
ZjAGO8	Forward: CGTATGCACCAACTACGCAA<br>Reverse: ATTGCAAACCACTGCCTAGC
ZjAGO9	Forward: CAACGACCGAAAGAACGGAA<br>Reverse: TCCTGTGGACAATGGATGGA
Actin	Forward: CTTGCATCCCTCAGCACCTT<br>Reverse: TCCTGTGGACAATGGATGGA
