# Supplementary material for: Infection with Jujube Witches’ Broom Phytoplasma Alters the Expression Pattern of the Argonaute Gene Family in Ziziphus jujuba
Source: Microorganisms. 2025 Mar 14;13(3):658. doi: 10.3390/microorganisms13030658 (PMC11944418; doi:10.3390/microorganisms13030658)
Supplement: Supplementary file 1 [file microorganisms-13-00658-s001.zip › Table S2.pdf]

Table S2. Primer design for gene clone and subcellular localization assay

Gene name	Primer Sequence (5'-3')
ZjAGO3	Forward: ATGCCTATAAGGCAAATGAAAGAGA<br>Reverse: ACAGTAAAACATCACCCTCTTCACA
ZjAGO6	Forward: ATGGATTCTGGTGAGCCTAATGGAA<br>Reverse: ACAGAAAAACATGGAACTGGACACG
ZjAGO9	Forward: ATGGAAGAGACAGAAGAGTCCAATG<br>Reverse: AGGCAAGGCCTTAGGAGGGGCAGGT
pSAK277-ZjAGO3-GFP	Forward: GTGGATCCAAAGAATTCATGCCTATAAGGCAAATGAAAGAGA<br>Reverse: CTCCTTTACCCATGAATTCACAGTAAAACATCACCCTCTTCACA
pSAK277-ZjAGO6-GFP	Forward: GTGGATCCAAAGAATTCATGGATTCTGGTGAGCCTAATGGAA<br>Reverse: CTCCTTTACCCATGAATTCACAGAAAAACATGGAACTGGACACG
pSAK277-ZjAGO9-GFP	Forward: GTGGATCCAAAGAATTCATGGAAGAGACAGAAGAGTCCAATG<br>Reverse: CTCCTTTACCCATGAATTCAGGCAAGGCCTTAGGAGGGGCAGGT
SAK	Forward: CATCGAAAGGACAGTAGAAAAGG<br>Reverse: CATTAGAATGAACCGAAACCG
